# Perceptual Oscillation of Audiovisual Time Simultaneity

**DOI:** 10.1523/ENEURO.0047-18.2018

**Published:** 2018-05-25

**Authors:** Alessandro Benedetto, David Charles Burr, Maria Concetta Morrone

**Affiliations:** 1 Dipartimento Di Ricerca Traslazionale e Delle Nuove Tecnologie in Medicina e Chirurgia, Università Di Pisa, via San Zeno 31, Pisa, 56123, Italy; 2 Istituto Di Neuroscienze, Consiglio Nazionale Delle Ricerche (CNR), via Moruzzi 1, Pisa, 56124, Italy; 3 Dipartimento Di Psicologia, Farmacologia e Salute Del Bambino (NEUROFARBA), Università Di Firenze, via San Salvi 12, Firenze, 50135, Italy; 4 IRCCS Stella-Maris, Viale Del Tirreno 331, Pisa, 56018, Italy Calambrone

**Keywords:** Audiovisual, Behavioral Oscillations, Decision Bias, Simultaneity, Theta, Time Perception

## Abstract

Action and perception are tightly coupled systems requiring coordination and synchronization over time. How the brain achieves synchronization is still a matter of debate, but recent experiments suggest that brain oscillations may play an important role in this process. Brain oscillations have been also proposed to be fundamental in determining time perception. Here, we had subjects perform an audiovisual temporal order judgment task to investigate the fine dynamics of temporal bias and sensitivity before and after the execution of voluntary hand movement (button-press). The reported order of the audiovisual sequence was rhythmically biased as a function of delay from hand action execution. Importantly, we found that it oscillated at a theta range frequency, starting ∼500 ms before and persisting ∼250 ms after the button-press, with consistent phase-locking across participants. Our results show that the perception of cross-sensory simultaneity oscillates rhythmically in synchrony with the programming phase of a voluntary action, demonstrating a link between action preparation and bias in temporal perceptual judgments.

## Significance Statement

Judgments of the temporal order of a visual and auditory stimulus are not constant over time but fluctuate rhythmically in the theta range (∼7 Hz) in synchrony with the execution of a voluntary movement. Interestingly, these oscillations precede the action execution by about half a second, indicating active synchronization between temporal criterion and the intention to move. Overall, the results point to the presence of a single shared clock for perception and action.

## Introduction

Action and perception are tightly coupled systems requiring strong coordination and synchronization over time. However, how the brain achieves synchronization is still a matter of debate. Several electrophysiological studies have shown that neural oscillations preceding sensory stimulation are causally linked to perception (VanRullen and Koch, 2003; Linkenkaer-Hansen et al., 2004; Busch et al., 2009; de Lange et al., 2013; Hanslmayr et al., 2013; Sherman et al., 2016; VanRullen, 2016), and that endogenous oscillations effectively shape perceptual processing. Stimulus-locked behavioral oscillations have been demonstrated in perceptual performance (Fiebelkorn et al., 2011; Landau and Fries, 2012; Romei et al., 2012; Ho et al., 2017), confirming that the system can lock activity to relevant environmental cues. A growing body of scientific literature has shown that voluntary actions can also synchronize perceptual rhythms: for up to a second before and after executing a voluntary action, some visual properties, including visual contrast sensitivity (Tomassini et al., 2015; Benedetto et al., 2016; Benedetto and Morrone, 2017), visual attention (Hogendoorn, 2016), or temporal integration/segregation (Wutz et al., 2016), oscillate at slow frequencies phase-locked with the action execution. Conversely, it has been shown that the phase of neural oscillations can predict reaction time to perceptual events (Lansing, 1957; Surwillo, 1961; Drewes and VanRullen, 2011), and that a transient visual response in the central nervous system can reset the phase of low-frequency tremor oscillations in peripheral muscles (Wood et al., 2015). Despite all this evidence connecting the intrinsic oscillatory nature of perception with action, there is no clear consensus about the basic mechanisms and function of rhythmic modulation on early brain function (Engel and Singer, 2001; Fries, 2005; Klimesch et al., 2007). One fascinating idea is that temporal mechanisms act through synchronization of endogenous oscillations, achieving in this way the temporal binding of independent perceptual, motor, and cognitive phenomena.

This suggestion is supported by recent electrophysiological experiments showing that the visual temporal windows of integration/segregation oscillate rhythmically within delta, theta, and alpha ranges (Ronconi et al., 2017), and similar effects have been shown for (multi-)sensory temporal resolution (Varela et al., 1981; Samaha and Postle, 2015; Cecere et al., 2015; Milton and Pleydell-Pearce, 2016; Benedetto et al., 2017). Moreover, EEG experiments have shown that brain rhythms in the delta/theta range can modulate both the predictability of event timing (Stefanics et al., 2010) and the encoding of memory sequences of events (Heusser et al., 2016).

It is well established that action and perceptual timing are strongly interconnected. Perceived visual duration can be strongly influenced by action, being compressed and/or dilated for perisaccadic stimuli, and also during hand movements (Haggard et al., 2002; Park et al., 2003; Morrone et al., 2005; Binda et al., 2009; Hagura et al., 2012; Tomassini and Morrone, 2016). This action-induced temporal modulation has been observed also for other sensory domains, such as tactile perception, suggesting that it is a general mechanism (Yarrow and Rothwell, 2003; Tomassini et al., 2012, 2014). To test directly if the temporal resolution of perceptual bias and sensitivity also fluctuate rhythmically, we measured the perceptual temporal order of auditory and visual targets before and after a voluntary action execution, hypothesizing a rhythmic modulation of perceptual bias, synchronized with the time of action.

## Materials and Methods

### Participants

Nine subjects (age mean ± SEM: 27 ± 0.6, 3 females) took part in the experiment. All had normal or corrected-to-normal vision and normal audition. All participants provided informed consent, and the experiment was approved by the ethics committee (Comitato Etico Pediatrico Regionale, Azienda Ospedaliero-Universitaria Meyer, Firenze, Italy).

### Apparatus

The visual stimuli were generated by the VisaGe (Cambridge Research System) controlled via CRS Toolbox for Matlab (Matlab r2007a, The Mathworks, inc.) and displayed at 57 cm on a gamma-calibrated CRT monitor (Barco Calibrator Line) with a resolution of 800 × 600 pixels, a refresh rate of 120 Hz, and mean luminance of 38.5 cd ⋅ m^−2^. Auditory stimuli were generated by the ViSaGe through its trigger output port, synchronized with the refreshing rate of the monitor, and amplified by a speaker positioned centrally, below the monitor. In this way, we ensured a perfect temporal synchronization between the visual and the auditory stimuli, verified by direct measurements. The responses were recorded with an infrared CB6 Response Box (Cambridge Research System) controlled via CRS Toolbox for Matlab.

### Stimulus and procedure

Participants maintained fixation on a red square (0.25°) presented in the center of screen on a gray background. The fixation appeared at the beginning of the session and lasted until the end. Stimuli consisted of a visual stimulus, preceded or followed by an auditory stimulus. The visual stimulus consisted of a black Gaussian-blob (contrast of 50%, sigma of 5°) presented in the center of the screen. The auditory stimulus was a short suprathreshold noise burst presented from a central speaker positioned below the monitor. Both visual and auditory stimuli were 8-ms duration and were synchronized with the refreshing rate of the monitor.

The task was a two-alternative forced-choice audiovisual temporal-order judgment (TOJ). Volunteers initiated the trial sequence by pressing a start button, and after a random delay from the button-press, the audiovisual sequence was presented. The stimulus-onset asynchrony (SOA) between the audiovisual stimulus was random, within 0 and ±500 ms (mean ± SEM trials per SOA: 74 ± 8), with a higher proportion of trials presented around the time of the physical simultaneity of the audiovisual stimuli (SOA between ±16 ms: 206 ± 57). The delay between the first stimulus of the pair and the button-press was randomly selected from 15 values: 0, 25, 50, 75, 100, 125, 150, 175, 200, 225, 250, 300, 350, 450, and 500 ms. Note that this sampling created a time resolution of 25 ms for the first 250 ms from button-press, decreasing to 50 ms for later latencies. Subjects had to report, via button press, whether the sound appeared to precede or succeed the Gaussian blob. The timing of the start of each trial and the responses were voluntarily paced, but had to be continuously spaced within 1.5–3.0 s, resulting in a continuous slow rhythmical tapping within 0.33 and 0.66 Hz. The trials that did not fit this timing were discarded from further analysis. To investigate the dynamics of audiovisual integration before action execution, in ∼20% of the trials the stimulus was unpredictably presented before the start signal. We asked participants to maintain their start/response pacing, even when the start action was not causally related to stimulus onset, but to continue to pay the same attention to these trials. Several sessions were recorded over different days. Overall, we collected 10,776 trials (1197 ± 257 trials per participant, mean ± SEM).

### Data analysis

**Figure 1.**
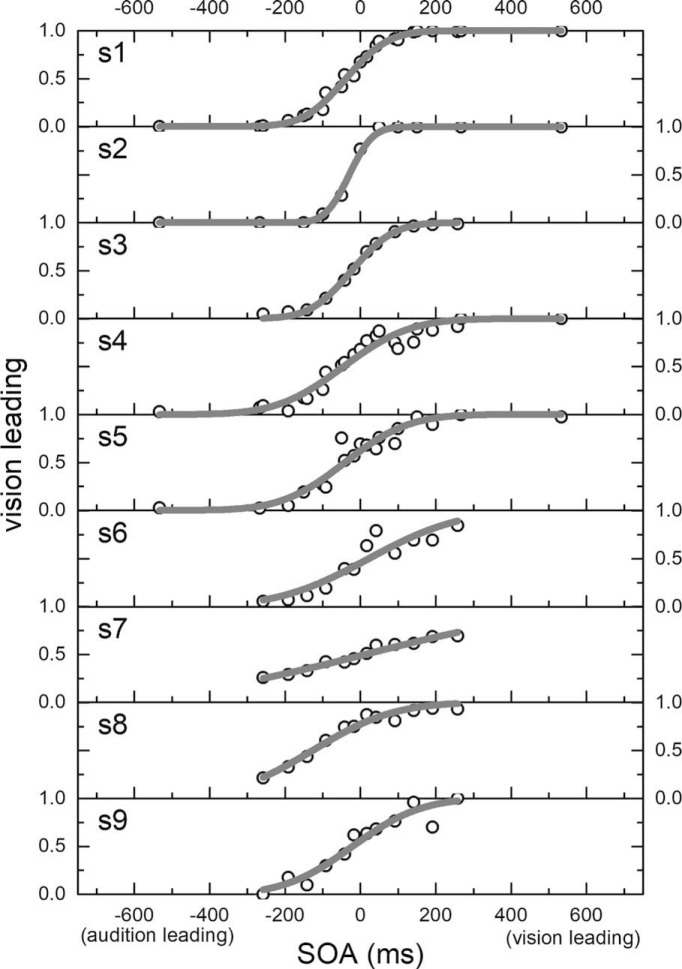
Data and fitted psychometric functions for all participants (*N* = 9) pooling all trials at all delays (1197 ± 257 trials per participant, mean ± SEM). Proportion of visual leading responses is plotted as a function of stimulus onset asynchrony (SOA), defined as positive when vision leads. Gray curves show the fitted psychometric function.

Analyses were conducted both on individual data and also after pooling all data together into a single dataset (hereafter termed the aggregate-observer). To investigate the bias and sensitivity dynamics, aggregate-observer responses were fitted with a cumulative Gaussian. The responses were reported as proportion of vision leading, modeled as a function of the signed SOAs of the auditory stimulus with respect to the visual. Perceptual bias was evaluated by measuring the point of subjective simultaneity (PSS) of the psychometric function (the point where the proportion of vision-leading is equal to 50%). Sensitivity was evaluated by the slope of the psychometric function, which corresponds to the just-noticeable difference (JND). This is a standard procedure following the Green and Swets signal detection theory (Green and Swets, 1966). An example of this fitting is shown in Fig. 1 for each individual observer. If not otherwise stated, the time from action refers to the temporal delay between the action onset and the first stimulus presented.

**Figure 2.**
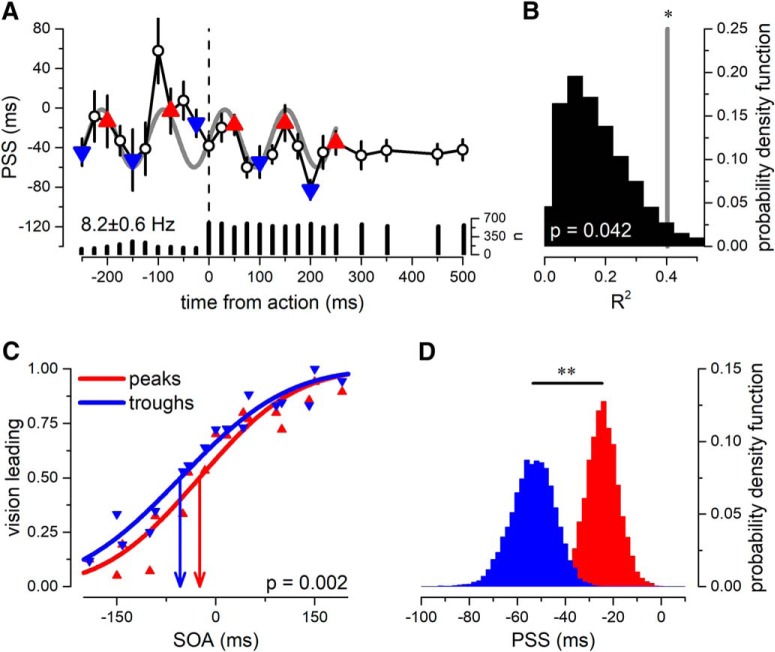
Dynamics of PSS for the aggregate observer (*N* = 9). ***A***, PSS ± 1 SEM as a function of time from action execution. The gray line reports the best sinusoidal fit, with a frequency of 8.2 Hz. Red upright triangles, points nearest to the predicted peaks of the oscillation; blue inverted triangles, points nearest the troughs of the oscillation. ***B***, Probability density function of the goodness of fit obtained by fitting the random shuffled data with its best sinusoidal fit. The vertical gray line reports the goodness of fit of the PSS oscillatory model being higher than what expected from chance (*p* = 0.042). ***C***, Proportion of vision-leading responses as a function of SOA, pooling together all trials falling within the peak (red triangles) or trough (blue triangles) of the best-fit oscillation in ***A***. Thick curves show the psychometric function computed on the datasets; vertical lines report the PSS for the peak and trough trials, in red and blue, respectively. ***D***, Bootstrap distribution (10,000 simulations, with replacement) of PSS for peak (red) and trough (blue) trials. The bootstrap *t* test revealed a significant difference between the two distributions (*p* = 0.002). Asterisks mark the statistical difference between the two distributions (*p*-value: 0.01 > ** > 0.001).

**Figure 3.**
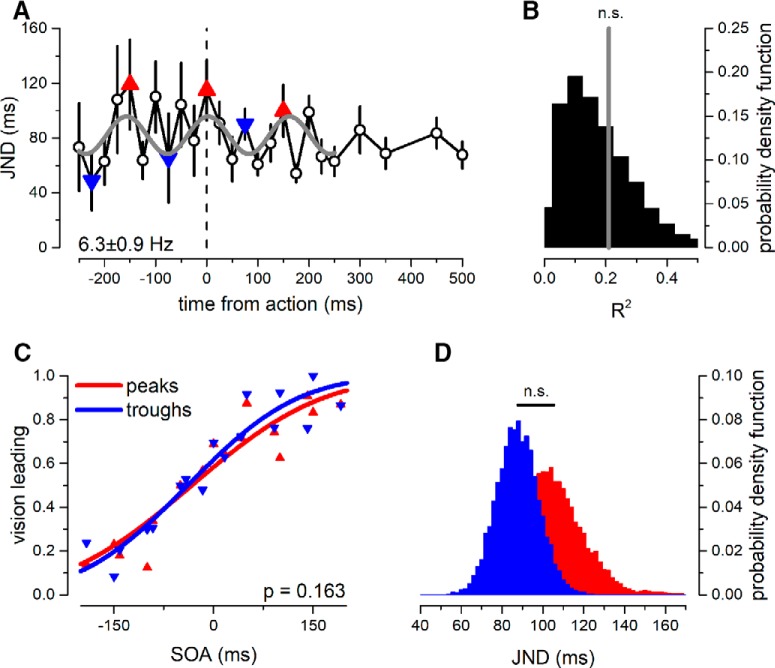
Dynamics of JND for aggregate observer (*N* = 9). ***A***, JND ± 1 SEM as a function of time from action-execution. The gray line reports the best sinusoidal fit, of frequency 6.3 Hz. Red upright triangles, points nearest to the peaks of the oscillation; blue inverted triangles, points nearest to the troughs of the oscillation. ***B***, Goodness-of-fit probability distribution obtained by fitting the random shuffled data with its best sinusoidal fit. The gray line reports the goodness of fit of the JND oscillatory model that does not exceed the chance-level threshold (*p* = 0.3). ***C***, Proportion of vision-leading response as a function of SOA, pooling together all trials falling within the peak (red triangles) or trough (blue triangles) of the best-fit oscillation in ***A***. Thick curves show the psychometric function computed on the datasets. ***D***, Bootstrap distribution (10,000 simulations, with replacement) of JND for peak (red) and trough (blue) trials. The bootstrap *t* test revealed that difference between the two distributions was not significant (*p* = 0.163).

We computed a psychometric function on the aggregate observer for each time lag from −275 to 500 ms from action execution (binning the pre-action trials in nonoverlapping bins of 25 ms) and extracted the PSS and the JND from each estimated model (Figs. 2 and 3). For both PSS and JND dynamics, we fitted the best sinusoidal model for each dataset at lags between −275 and 250 ms from action execution. We selected the nearest bins falling around the time of a peak or trough of the best oscillation and computed a psychometric function pooling together all trials falling the peak or trough bins. A bootstrap *t* test was run to test differences in PSS and JND between the peaks and the troughs of the sinusoidal model. The best-fit oscillatory models from the PSS and the JND were statistically evaluated with a bootstrap procedure on surrogate data obtained by randomly shuffling the responses of each trial (using the same time stamps), then performing the standard binning procedure (1000 reiterations). To control for multiple comparisons, the surrogate data were fitted with the best sinusoidal wave form, with frequency, amplitude, and phase as free parameters (Benedetto and Morrone, 2017). A one-tailed nonparametric bootstrap *t* test was run to assess whether the *R*-squared values of the best fit of the data were statistically higher than the 95% of the *R*-squared distribution obtained from the surrogate dataset.

To test the spectral content of the vision leading dynamics (as an approximation of response bias), we ran a single-trial multivariate generalized linear model (glm) analysis (Tomassini et al., 2017) on the aggregate observer for lags within −450 and 250 ms from action execution, including all trials with an SOA less than ±100 ms. No bias was present in the distribution of the SOAs across subjects and time-from-action execution (mean SOA ± interquartile range: 0.26 ± 3.05 ms). We fitted a linear regression model including as predictors a sine and a cosine for a given frequency of interest $f_{i}$. The probability model behind this analysis can be written as

$${\hat{Y}}_{n}=\beta_{0}+\beta_{1}\text{sin}\left(\omega t_{n}\right)+\beta_{2}\text{cos}\left(\omega t_{n}\right),$$

where t is the time lag of the single trial n; $\beta_{0}$, $\beta_{1}$, and $\beta_{2}$ are the fixed-effect linear regression parameter;${\hat{Y}}_{n}$ is the predicted behavioral performance; and ω is the angular frequency ($\omega =2\pi f_{i}$). The fixed-effect linear regression parameters were estimated using standard least square method (LSM) as

$$\left[\begin{array}{c} \beta_{0}\\ \beta_{1}\\ \beta_{2} \end{array}\right]={\left(X^{t}X\right)}^{-1}X^{t}Y,$$

where Y is the vector of the single trial responses (0 or 1 for audition or vision leading responses, respectively). The matrix X has the form:

$$X=\left[\begin{array}{c} 1\\ \ldots \\ 1 \end{array}\begin{array}{c} \sin\left(\omega t_{1}\right)\\ \ldots \\ \sin\left(\omega t_{n}\right) \end{array}\begin{array}{c} \cos\left(\omega t_{1}\right)\\ \ldots \\ \cos\left(\omega t_{n}\right) \end{array}\right].$$

Statistics were computed via permutation obtained by shuffling the responses (10,000 repetitions) to create an empirical noise distribution. We adopted the maximum statistics to correct for multiple comparison: for each permutation, we selected the maximal amplitude of the beta coefficients across all frequencies and ran a bootstrap *t* test between the distribution of the maximal amplitudes and the amplitude obtained from the actual dataset for each frequency. The spectral amplitude (*A*) was computed as the Euclidean length of the $\beta_{1}$ and $\beta_{2}$ coefficients as

$$A=\sqrt{\beta_{1}{}^{2}+\beta_{2}{}^{2}}.$$

The amplitude error was estimated by implementing a jackknife resampling procedure to explore the weight of each participant in determining the actual result.

The advantage of this analysis over more traditional approaches (e.g., Fourier analysis or fitting) is that there is no need to bin the data, which can potentially give rise to spurious results given the fixed constant sample time. The analysis also works well for nonuniform and sparse samples, and for this reason is optimal not only for aggregate observer analysis, but also for group mean statistics. We investigated a frequency range between 3 and 15 Hz (with a resolution of 0.01 Hz). Furthermore, we selected the peak of significance from the aggregate observer analysis to investigate single-subject variability. For the selected frequency, we tested against zero the average of the participant-specific beta coefficients (expressed as amplitude and phases of the significant components) by means of the bivariate Hotelling’s *T*-squared statistic (as in Tomassini et al., 2017):

$$T^{2}=k{\left({\bar{\beta }}_{1,}{\bar{\beta }}_{2}\right)}^{t}S^{-1}\left({\bar{\beta }}_{1},{\bar{\beta }}_{2}\right),$$

where k denotes the number of subjects, $\left({\bar{\beta }}_{1},{\bar{\beta }}_{2}\right)$ is the sample mean (across subjects) of the vector of linear regression coefficients $\left(\beta_{1},\beta_{2}\right)$, and $S^{-1}$ is the inverse of the sample covariance matrix of these vector-valued regression coefficients. This Hotelling’s *T*-square test is an extension of the Student’s *t* test to the multivariate domain, and it provides significant results only if the regression coefficients are large and have the same sign across subjects (Tomassini et al., 2017).

We also investigated group-mean fluctuations in bias with glm analysis. We applied the analysis to each individual subject, and then averaged—for each tested frequency—the norm of the resulting sine and cosine vector (norm of vector average). It is noteworthy that this approach, similarly to the aggregate observer analysis, is aimed at revealing the phase-locked oscillations across participants, the modulation of the bias that is shared by all subjects. The error of the spectrum was computed via a jackknife resampling. Additionally, to further assure us that the significant oscillation was restricted to those frequencies with higher across-subject coherence, the Hotelling *T*-squared test was extended to all the tested frequencies (3–15 Hz).

## Results

Our experiment investigated the bias and sensitivity dynamics in a multisensory TOJ task. Fig. 1 shows the psychometric functions for the TOJ task (proportion of trials where vision was seen as leading, as a function of SOA), for all participants (*N* = 9), computed pooling together all trials at all delays from button press (go signal). The slopes of psychometric functions were shallow (JND mean and SEM, 105 ± 24 ms; interquartile range, 58 ms), suggesting the task was demanding for participants and sensitivity poor as previously observed (Arrighi et al., 2006; Binda et al., 2009). Consistent with previous results, participants showed a weak but consistent bias toward “vision first,” with average negative points of subjective synchrony (PSS mean and SEM, −33 ± 14 ms; interquartile range, 29 ms).

Fig. 2*A* shows the dynamics of PSS as a function of time from action. The PSS was not constant but fluctuated rhythmically as a function of time-from-action, at a frequency of 8.2 Hz, with amplitude of ±29 ms. A time difference of ∼50 ms of subjective simultaneity is a large effect for audiovisual signals (Fujisaki et al., 2004). Clear oscillations emerged for 500 ms around action-execution, then attenuating in amplitude. To evaluate the significance of the sinusoidal fit, we compared the *R*-squared values of the best fit with the distribution of *R*-squared obtained by fitting the best sinusoidal wave form to surrogate data. Fig. 2*B* shows that the goodness of fit of the PSS model was statistically higher than that expected from a noise distribution (*R*-squared = 0.4, *p* = 0.042).

As a further test of the significance of the modulation, we selected all the trials in the bins at the predicted peaks or troughs of the best-fitting oscillation (Fig. 2*A*). As shown in Fig. 2*C*, the two independent psychometric functions, one for each selected dataset, had a difference in PSS of ∼30 ms, but the same slope (i.e., sensitivity). The difference in PSS was significant (*p* = 0.002, bootstrap sign-test with 10,000 reiterations; Fig. 2*D*).

Fig. 3*A* reports the dynamics of the JND of the psychometric function, again as a function of time from action-execution. No significant sinusoidal fit was obtained: the best fit was at 6.3 Hz, but this was not significant (amplitude = 13 ms; R-squared = 0.21, *p* = 03; Fig. 3*B*). Even after pooling all trials of the bins nearest to the predicted peak or trough of the best-fit oscillation, there was no significant difference in the slope of the psychometric functions (*p* = 0.163; Fig. 3*D*).

**Figure 4.**
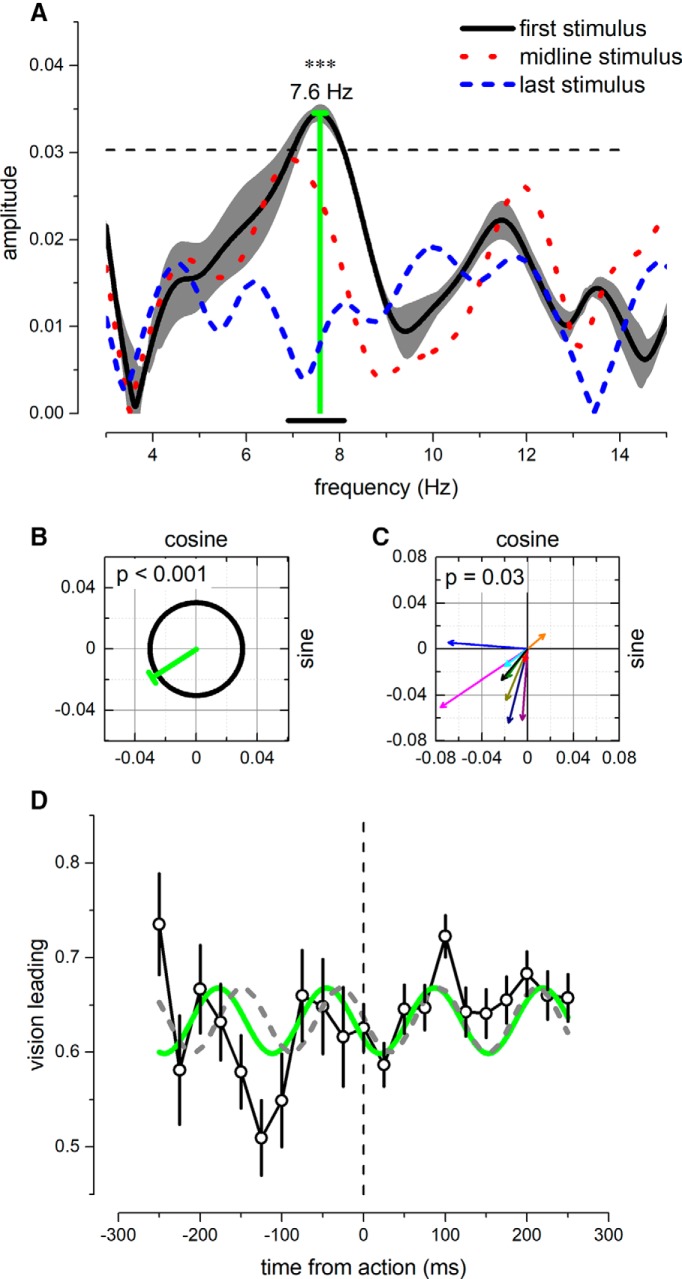
***A***, Multivariate glm analysis for the aggregate observer (for the interval range within –450 and 250 ms from action execution). The thick black curve shows the amplitude modulation as a function of frequency, for trials aligned to the first stimulus; the gray shadow reports the interquartile range of the amplitude error estimated with a jackknife resampling; dotted red and dashed blue curve represent the amplitude for trials aligned to midline or last stimulus, respectively. Only frequencies ∼7.6 Hz for trials aligned to the first stimulus were significant (vertical green line; uncorrected *p* < 0.001, corrected *p* = 0.013). Horizontal dashed line marks the 95th percentile of noise distribution computed via the maximum statistics for permuted data. ***B***, Phase distribution of the strongest frequency above noise level, at 7.6 Hz. Green and black lines report the norm of the betas computed for 7.6 Hz and the 95th percentile of noise distribution calculated for the best sinusoidal fitted frequency for each permutation (see Methods). ***C***, Results from the group-mean analysis computed for the aggregate observer’s significant frequency of 7.6 Hz. Colored arrows report single subjects’ beta distribution (sine and cosine); black arrow shows the group-mean betas. The vectors scatter around the third quadrant, suggesting strong phase-coherence across participants. The Hotelling *T* ^2^ revealed that the beta distributions were statistically significant (*p* = 0.03). ***D***, Proportion (±1 SEM) of vision leading response as a function of time from action (*N* = 9). Gray curve shows the reverse best-fit sine model estimated on the PSS, from the dataset of Fig. 2*A*; green curve reports the function obtained by the multivariate glm methods shown in ***A***.

We further investigated the frequency spectrum of the bias dynamics with a single-trial multivariate glm analysis, which allowed us to eliminate the binning procedure. As an indirect index of bias, we computed the proportion of “vision leading” responses as a function of time from action (Fig. 4*D*), for all SOAs between visual and auditory stimuli less than ±100 ms and within a temporal range of −450 to 250 ms from action execution (see Methods). Fig. 4*A* reports the result of this analysis for the aggregate observer, run on the probability of reporting “vision leading,” with time aligned with the timing of the first stimulus of the audiovisual pair (black thick curve), the middle of the pair (red dotted curve), or the last (blue dashed curve). When aligning the action with the presentation of the first stimulus, PSS showed strong and statistically significant theta oscillations at 7.6 Hz (amplitude and phase: 0.034 and 216°; *p* = 0.012, corrected for multiple comparison: see Methods). When the same analysis was run with responses aligned to the onset of the last stimulus, or with the stimulus midline, no statistical significant oscillations emerged in the PSS (*p* > 0.05, corrected for multiple comparisons). This suggests that the oscillations in PSS modulate the time of the first sensory event.

The results obtained from the multivariate glm analysis on single trials are consistent with those analyzing the best fits of the binned time-series. The two methods predicted similar sinusoidal oscillations in frequency, amplitude, and phase as shown by the gray and green curves of Fig. 4*D*, showing the separate estimates of predicted oscillations: gray line, best-fit analysis, adapted from Fig. 2*A*; green line, single-trial frequency analysis, from Fig. 4*B*). Both functions fit well the oscillation of the proportion of trials reported as “vision leading,” calculated from the independent bins of 25 ms.

Finally, we performed an additional control analysis on single subjects to test whether the aggregate-observer results could have been driven by a few participants with strong phase-locked oscillations in bias. We selected the frequency of 7.6 Hz, suggested by the aggregate observer analysis (Fig. 4*A*). For each subject, we estimated the sine and cosine components via glm analysis and computed the Hotelling *t*-squared statistic to assess the statistical significance of the beta distribution (see Methods). Fig. 4*C* shows the results of this analysis. Confirming and validating the aggregate-observer results, we found a consistent phase-locking within subjects at ∼7.6 Hz (Hotelling *t* ^2^_8_ = 12.6, *p* = 0.036). The average amplitude and phase of the individual subject’s vectors at 7.6 Hz were 0.035 and 228°, respectively, very similar to that of the aggregate observer. Globally, these results indicate the presence of a strong theta oscillation in bias for audiovisual TOJ, synchronized with the action onset and encompassing it, showing very similar temporal dynamics across participants.

**Figure 5.**
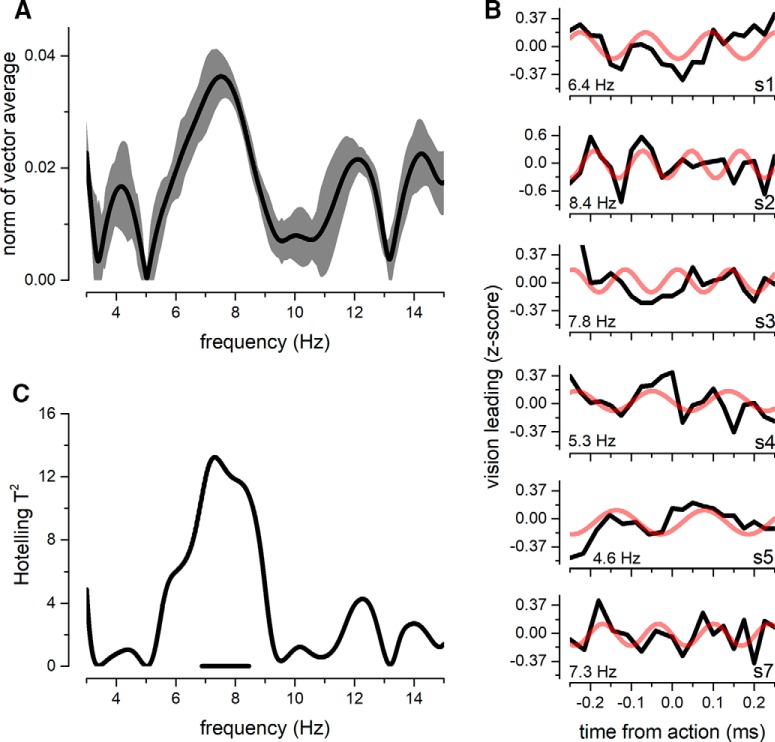
***A***, Norm of the group-mean average vector obtained for the proportion of vision leading time courses. Shaded gray area reports the interquartile range obtained from jackknife resampling procedure. ***B***, Single-subject fluctuations in *z*-scored vision leading response for six representative subjects. The red line shows the best sinusoidal fit for each individual participant. ***C***, Hotelling *T*-squared distribution for all the tested frequencies. The only significant spot of coherence is ∼7.6 Hz. The horizontal thick line reports the significant frequencies (*p* < 0.05).

To better assess the interindividual differences, we also performed a glm analysis on single subjects. The spectrum of the group-mean analysis is reported in Fig. 5*A*, which shows, for each tested frequency, the length of the mean vector across participants. Confirming the aggregate-observer result, the spectrum shows a peak at ∼7.6 Hz, indicating a general increase in phase coherence for that frequency. To further confirm the presence of a single oscillatory spot of modulation in the bias, the Hotelling *t*-squared statistic was extended to all possible frequencies (Fig. 5*C*). The *t*-squared distribution confirms the aggregate-observer results, indicating a group-mean phase locking at ∼7.6 Hz only. Fig. 5*B* additionally shows single subject modulations of the *z*-scored proportion of visual leading responses for 6 representative subjects. These plots confirm the well-known subject-by-subject variability previously reported for behavioral oscillations (Fiebelkorn et al., 2013; Tomassini et al., 2015; Ho et al., 2017), but nevertheless show similar oscillatory modulation for each participant. The best sinusoidal fit revealed a mean frequency and interquartile range of 6.6 ± 2.5 Hz, with *R*-squared of 0.269 ± 0.13. Importantly, it is fundamental to point out that despite the overall interindividual differences, our analysis clearly shows a consistent phase-locked oscillation in bias, for all 9 participants.

## Discussion

We measured sensitivity and bias dynamics in temporal order judgment of transient audiovisual stimuli presented around the time of executing a voluntary action (i.e., button-press). We found that audiovisual temporal bias oscillates in synchrony with action-execution, in the high-theta range (at ∼7–8 Hz), while no significant oscillations were observed for sensitivity of temporal order. The oscillations in bias were phase-locked with the first stimulus of the temporal sequence, not with the second or the barycenter of the two. The temporal rhythmicities commence ∼500 ms before action-execution and last for up to 250 ms after button-press, with strong phase coherence across subjects. Interestingly, the phase of the prominent theta oscillation in bias reveals a continuous dynamic that encompasses the action onset, with no phase-reset or frequency modulation at the actual action onset, suggesting a close link between the programming signals that precedes action-execution and perceptual processing.

Despite the strong phenomenological impression of a unitary sense of time, it is not uncommon for the brain to deal with multiple processing speeds in its computations: time has been shown to vary across sensory modalities and features of the sensory stimulation (Johnston et al., 2006; Kanai et al., 2006; Burr et al., 2011; Harrington et al., 2011; Tomassini et al., 2011). Considering all these modality- and input-specific clocks or oscillators in the brain, how do we achieve a stable and unitary sense of time? It has been shown that time perception and motor timing rely on similar cerebral structures (Schubotz et al., 2000; Nobre and O’Reilly, 2004), suggesting that motor systems play a key role in shaping a unitary sense of time, possibly by synchronizing the dynamics of local processing. According to this hypothesis, brain oscillations strongly correlate with time perception (Varela et al., 1981; Large and Jones, 1999; Barnes and Jones, 2000; Herrmann et al., 2013; Samaha and Postle, 2015; Benedetto et al., 2017; Ronconi et al., 2017).

Recent electrophysiological and behavioral experiments have shown that the motor system and perceptual rhythms are synchronized over time (Gaarder et al., 1966; Tomassini et al., 2015, 2017; Benedetto et al., 2016; Benedetto and Morrone, 2017; Hogendoorn, 2016; Tomassini and D’Ausilio, 2018; Wutz et al., 2016). Neural oscillations have been proposed to reflect ongoing modulation of cortical excitability (Klimesch et al., 2007; Jensen and Mazaheri, 2010; Jensen et al., 2012, 2014). In agreement with this hypothesis, several studies have shown that ongoing oscillations in the theta-alpha range are related to fluctuations in perceptual sensitivity across different modalities (Ng et al., 2012; Busch et al., 2009; Fiebelkorn et al., 2013; Milton and Pleydell-Pearce, 2016; Craddock et al., 2017). It has been demonstrated that cross-modal interactions are also achieved by synchronization of cortical neural oscillations in the delta, theta, and gamma frequencies (Lakatos et al., 2007), and that rhythmic synchronization between different brain areas might serve to bind cross-modal information (Senkowski et al., 2008).

In our experiment, we investigated audiovisual temporal order judgments, reporting oscillations in perceptual bias—in the theta range—phase-locked with the time of action-execution (button-press). Theta oscillations have been proposed to mediate human anticipatory mechanisms (predictive priors), by modulating faster neuronal synchronization and facilitating neuronal communication among distant brain areas (Lakatos et al., 2008; Stefanics et al., 2010). A recent experiment has shown that the phase of the prestimulus occipital alpha oscillations can predict the subjects’ perceptual decision in a detection task (Sherman et al., 2016): the oscillations in temporal bias reported here may indeed reflect rhythmic, top-down influences of perceptual predictions, modulating our decisions according to the timing of action execution. It is noteworthy that (in our paradigm) we are able to record behavioral oscillations only if these rhythmicities are phase-locked with the motor execution. The results show not only that perceptual decisions oscillate in synchrony with the button-press, but also that these oscillations precede the action execution (by about half a second), indicating a synchronization between our perceptual decisions and the intention to move. Furthermore, the strong phase-coherence reported across participants suggests not only that this oscillatory dynamic is very precise, but also that it is likely to reflect a general sensorimotor rhythm in the brain, pointing to the presence of a single shared clock driving both perception and action.

The dynamics of the modulation in criterion shows a local maximum of decision bias just before and after the action execution (Figs. 2*A* and 4*D* , as indicated by maximal distance between the oscillatory model and the ideal no-bias point), with reduced perceptual bias around the time of action-execution (0–100 ms from button-press). We speculate that this precise sensorimotor coordination has the goal of minimizing prior interference around the time of action-execution, when the subject is actively interacting with the environment and likely to be producing perceptual events. On the contrary, during action planning and motor-induced suppression periods (around −50 and 100 ms from button press, respectively), the weight of priors seems to be maximum, resulting in a stronger bias. Furthermore, these oscillations were phase-locked with the first stimulus of the sequence; no oscillations were observable when the action was aligned with the last or the midline stimulus. This fact serves as an important sanity check for the robustness of our spectral analysis, as well as speaking to the dynamics of the oscillatory process under description. The results are consistent with the idea that oscillations in bias mostly affect the first stimulus, which may in turn induce a reorganization of local-global brain oscillations (Romei et al., 2012).

We observed oscillations in temporal bias, but no oscillations in temporal sensitivity. The scientific literature on perceptual cycles has mainly focused on the effect of oscillations on sensitivity and reaction time (VanRullen, 2016). However, a recent experiment has shown that sensitivity is not the only perceptual feature that exhibits rhythmic dynamics, but that decision criteria also oscillate (Ho et al., 2017). Crucially, the two dynamics seem to be largely independent, oscillating at different frequencies and phases (Ho et al., 2017), suggesting the presence of two distinct driving mechanisms shaping the rhythms of perception. This idea is supported by several electrophysiological studies showing that alpha and theta oscillations are instrumental in transmitting feedback signals in the brain (Pastoll et al., 2013; van Kerkoerle et al., 2014; Bastos et al., 2015; Jensen et al., 2015; Andreou et al., 2017). It has also been shown that alpha phase and power can specifically predict decision (Sherman et al., 2016; Benwell et al., 2017; Craddock et al., 2017). Our results are consistent with the idea of two separated oscillatory mechanisms for sensitivity and bias. Sensitivity oscillation may be linked with local modulations of cortical excitability (Klimesch et al., 2007; Jensen and Mazaheri, 2010; Jensen et al., 2012, 2014), while decision criteria oscillations may be driven by top-down predictive mechanisms (Sherman et al., 2016; Ho et al., 2017). We further show here that oscillations in decision can be synchronized with voluntary action, emerging around half a second before executing a movement. However, there is still no conclusive evidence about the causal direction of the synchronization between action and perception: either the intention-to-move signal arrives in perceptual areas and synchronizes oscillations there; or both perceptual and motor actions are gated by an endogenous neural rhythm. Both mechanisms are equally possible.

To conclude, we demonstrate that audiovisual temporal bias, but not sensitivity, oscillates in the theta range, synchronized with action execution. These oscillations are instantiated hundreds of milliseconds before the movement onset, indicating that the organization of a voluntary action is directly linked to the way we process sensory information about temporal order. These rhythmic influxes are very precise in time and similar across subjects, suggesting a fundamental functional role in shaping our criterion over time and pointing to the presence of a single shared clock between perception and action.

## Synthesis

Reviewing Editor: Bradley Postle, University of Wisconsin Decisions are customarily a result of the Reviewing Editor and the peer reviewers coming together and discussing their recommendations until a consensus is reached. When revisions are invited, a fact-based synthesis statement explaining their decision and outlining what is needed to prepare a revision will be listed below. The following reviewer(s) agreed to reveal their identity: Vincenzo Romei

Because the two reviews are largely in accord (although the emphasize different points), I am simply pasting them in here:

Reviewer #1

This paper measures audio-visual perceptual simultaneity at different lags relative to a motor action (button press). The lags are sampled with high temporal resolution (25ms bins), allowing the authors to analyze the resulting time-varying signals in terms of spectral content. They find that the bias in perceptual judgments (the preference to report visual or auditory stimuli as the first among the two) but not the sensitivity (the slope of the psychometric functions) oscillates at about 8Hz. Because the time reference for these oscillations is the moment of a button press, the authors conclude that motor actions and perceptual audio-visual simultaneity judgments are synchronized by a common 8Hz rhythm.

I find the approach and the conclusions interesting. There is one statistical issue that I believe still remains to be addressed. Beyond this, I mainly have requests for clarification.

1. My main issue is an apparent circularity in the demonstration of the oscillatory effect. The curve relating bias (or PSS) to temporal lag is fitted with an oscillation by trying all possible phase and frequencies values. An 8Hz oscillation is found to fit best, but of course, for any time-varying curve there will always be a best-fitting oscillation, so we really need statistics here to be sure that this oscillatory fit is higher than you would expect by chance. As far as I can tell the authors do not directly report this statistical test, but say (lines 161-162): “To test whether this modulation was significant, we selected all the trials in the bin at the predicted peak or trough of the best-fit oscillation” and basically go on to test whether the troughs are lower than the peak. That test, however, is ‘circular’ in the sense that it is biased towards significant outcomes by the fitting procedure. This could still be valid as a second step, e.g. to measure the magnitude of the PSS fluctuation, but it cannot count as an independent demonstration of the oscillatory nature of audio-visual simultaneity. The authors need a primary test to demonstrate this finding.

2. Although I understand the general approach, I was confused by the specific of the single-trial multivariate linear regression analysis. I believe some information is missing or some clarifications are needed. First, what is the dependent variable (Yn) in the equation on line 123? This should be stated explicitly. The only behavioral variable that is available on single-trials is the binary outcome (vision first/audition first) but the equation does not seem to model a binary process. Also, I am confused by the lack of any term representing the particular audio-visual SOA on that trial: surely this should influence the behavioral response at least as much as the temporal lag between stimuli and button press. I wonder if the authors are actually regressing the probability of ‘vision-first’ responses across the different temporal lag bins. If that is the case, then this is not a single-trial regression... Please detail and clarify.

3. The data indicate that the button press and the audio-visual simultaneity judgments are somehow synchronized or phase-locked. There is no logical reason, however, to favor any causal direction in the interpretation: it could be that motor rhythms influence simultaneity judgments, or that motor actions are preferably triggered on a specific phase of an ongoing perceptual rhythm affecting simultaneity judgments. The authors clearly favor the former conclusion (e.g. lines 18, 19-20, 213, 225, 246, 276, 279, 280), but that is not justified. A minima, I believe that all of these statements (and any equivalent ones) need to be complemented by ‘and vice-versa’. Ideally, the entire manuscript could be reframed in a more neutral way.

4. Lines 250-256, this description of changes occurring at specific delays (0-100ms from button press, then -50 and 100ms from button press) did not correspond to anything I could see in the actual data (neither in Figure 2A nor in 4C). This needs to be clarified.

Reviewer #2

In an audio-visual temporal order judgment task, the authors investigate the relationship between perception and action by looking at the oscillatory dynamics of temporal bias (and sensitivity) before and after the execution of voluntary hand movements. The authors show that the audio-visual sequence was rhythmically biased as a function of delay from hand movement at a pace typical of the theta range around the time of the hand movement, showing consistent phase-locking across participants.

The authors interpret these findings as evidence that time perception rhythmically oscillates in synchrony with the programming phase of a voluntary action. They conclude that the motor system plays a leading role of the in modulating our sense of time flow.  

This is an interesting paper with clear hypotheses and sound methods. The question posed is highly relevant. However there are a number of issues that I would like them to address.

1. A first issue is more conceptual in nature and concerns the interpretation of the temporal order judgment as directly related to explicit time perception. While there is increasing evidence showing how temporal resolution of sensory processes is strictly related to a discrete sampling of information, there is not yet demonstration of a direct link between time perception and oscillatory properties of sensorymotor processing. Nor this work explicitly account for time perception. The task relates to temporal order judgment: which stimulus comes first. At best, the task represents an implicit measure of time resolution, but not an explicit temporal reference to the perception of time passing. Therefore I would tone down the interpretation of current findings as explicitly referring to time perception rhythmically fluctuating in synchrony with voluntary action. Instead, the authors might want to refer to the temporal precision of perceptual bias (vs. sensitivity) to fluctuate in synchrony with voluntary action.

2. A second conceptual remark in the interpretation of the results concerns the conclusion that the motor system leads the modulation of our sense of time flow. This is an overstatement for the following reasons. First, this is not an explicit task on time perception, but on temporal order of sensory events, therefore any sense of time flow is not directly tested by the task. Second, the authors have locked their stimuli to a voluntary action and found this to nicely phase-lock temporal precision of perceptual bias that now cyclically oscillates at theta frequency. However, this is not enough prove that the motor system leads temporal precision (or even time flow). This is prove that the motor system is tightly linked to sensory processing and interacts with it. If the authors were to introduce another independent variable (instead of the voluntary button press), say a somatosensory stimulus over the same index finger, this might similarly lead to phase-locked sensory processing and show precision of perceptual bias to similarly fluctuate. This would clearly tone down the conclusion that the motor system leads the sense of time. However this measure is not available and therefore no definite conclusion can be reached in this sense. Instead, these interesting findings suggest that the organization of voluntary action directly impact the way we process sensory information by locking temporal perceptual bias to action preparation and control.

3. A third concern I have is related to the analysis performed. The authors state that they have conducted the analysis on individual data and by pooling data of all participants in a single dataset (referred to as aggregate observer). However, it becomes clear in what follows that the main analyses have been performed on the aggregate observer only. I am not persuaded this is the best way to analyse the data. The authors show the fitting on perceptual fluctuations as a function of button press for the aggregate observer, but do not report fitting for each of the 9 participants relative to movement onset. One would like to know the goodness of fit at the individual level. Importantly, one would like to see the variability on such analysis. It is not clear whether the 7.6Hz value is a spurious effect obtained by analysing the aggregate observer or it rather reflects the average impact across the 9 participants. One would like to see the same analysis performed on each participant and see how the aggregate observer analysis does not differ from the average of the individual fittings. Similarly, I wonder whether a lack of effect on JND fitting is similarly the result of a spurious calculation induced by the aggregate observer. Wouldn't the analysis on a single subject performance better return the veridical impact of action on temporal sensitivity? Wouldn't be the average of each individual fitting a better representation than the fitting of the average impact of motion on perceptual bias, where interindividual differences may have completely obscured the complexity of the underlying patterns?

The authors report at the end of the results section that they have tested at the individual level whether their results might be explained by some participant driving the effect at 7.6Hz. However the authors have not tested at the individual level whether the fitting at 7.6Hz is the best fitting for each of the 9 participants. All they show is that they somehow contribute significantly to the aggregate observer curve fitting oscillating at 7.6Hz. Nevertheless, this does not prove that the best fitting for each individual is otherwise faster or slower than the aggregate one, which might just be the result of a spurious combination. The authors should run the same analysis performed for the aggregate also for the individual datasets and pull together the averaged best fitting scores from each participant, thus better taking into account interindividual differences.

Alternatively, the authors should better explain why their approach is valid and valuable and preferable to the single subject procedure I propose to undertake here.

4. Finally, another important issue the authors should address is to provide a cleat conceptual and operational definition of response bias (or criterion) or perceptual bias on the one hand and sensitivity on the other hand. I would suggest to clearly define what they refer to as perceptual bias (and sensitivity), and uniform their description throughout the text. Importantly, they should offer the reader from very early how this conceptual definitions transfer of perceptual bias and sensitivity are operationalised in TOJ task. This does not come across clearly.

Other minor comments are reported below.

5. The authors refer to the concept of time perception in the introduction in a misleading way: “If neuronal time mechanisms are implicated in the synchronization, we may observe that also perceived time may oscillate rhythmically.” Thereafter they refer to temporal resolution of sensory processing rather than time perception per se. As described above, there is a confusion of different conceptual plans which need to be addressed throughout the manuscript.

6. The final sentence of the introduction is similarly misleading: “To test directly if the sense of time also fluctuates rhythmically, we measured the perceptual temporal order of auditory and visual targets before and after a voluntary action execution, hypothesizing a rhythmic modulation of perceptual bias, synchronized with the time of action.” The authors are not testing the sense of time, but rather the temporal resolution of perceptual bias. Again, as in previous comment, please address this issue throughout the text to avoid overstatements.

7. In the ‘significance statement’ the authors refer to ‘judgment of simultaneity’, but this is misleading as the task is a ‘temporal order judgment’, which relays at least in part to higher-level processes than simultaneity judgments. Please rephrase accordingly.

8. Discussion: similar issues as in the introduction. The authors should consider rephrasing their arguments relative to time perception in the light of the comments provided. Specifically, there is no direct evidence here that time perception has been altered by voluntary action. Rather, voluntary action seems to time-lock perceptual temporal bias which fluctuates in a rhythmic fashion at around 7-8 cycles per second.

9. The authors report in the discussion: “Finally, we reported oscillations in temporal bias, but no oscillations in temporal sensitivity.” As already commented above, they should better define at the beginning what they refer to with temporal bias and temporal sensitivity.

10. Please report the percentage of trials that did not fit the 1.5-3 sec timing of movement pace and that were discarded form the analysis.

11. The authors report to have recorded several sessions over different days. However it is not clear why a differential number of trials per participant has been collected. Also, on average, how many trials per SOA have been collected? This information might be helpful to appreciate the nature and strength of fitting of the data to the sinusoidal functions.

12. There are a number of typos, grammatical and syntax issues, but also issues with definition of concepts throughout the text. I suggest the authors to work on these aspect to improve readability. Below I report a non exhaustive list of issues I faced:

“ Please rephrase the following sentence in the introduction: ”

“Several electrophysiological studies have shown that neural oscillations preceding sensory stimulation are causally linked to perception (Busch et al., 2009; de Lange et al., 2013; Hanslmayr et al., 2013; Linkenkaer-Hansen et al., 2004; Sherman et al., 2016; VanRullen, 2016; VanRullen and Koch, 2003), showing endogenous oscillations effectively shape brain processing.”

‘Shown’ and ‘showing’ is redundant within the same sentence. Moreover, I would refer to perceptual rather than brain processes, as endogenous oscillations already represent brain processes.  

“ Please check grammar: Phase-locked ‘WITH’ or ‘TO’ the action execution?”

“ Please check grammar; in this sentence, for example: “This action-induced temporal modulations has been observed”.”

“ Check grammar: e.g. nine subject ‘take’ or ‘took’ part in the experiment?”

“ Check sentence: “The oscillations were strong from about −250 ms from action to 250 ms, thereafter greatly attenuating in amplitude””

“ It is not clear what the authors refer to as “Perceived visual time”.”

“ Please refer to Sensory domain, rather than Sensorial domain.  ”

“ Please spell out 2AFC.”

“  Please avoid terms as ‘highly statistically significant’.”
